# Aquablation therapy in large prostates (80–150 cc) for lower urinary tract symptoms due to benign prostatic hyperplasia: WATER II 3‐year trial results

**DOI:** 10.1002/bco2.121

**Published:** 2021-10-28

**Authors:** Kevin C. Zorn, Mohamed Bidair, Andrew Trainer, Andrew Arther, Eugene Kramolowsky, Mihir Desai, Leo Doumanian, Dean Elterman, Ronald P. Kaufman, James Lingeman, Amy Krambeck, Gregg Eure, Gopal Badlani, Mark Plante, Edward Uchio, Greg Gin, S. Larry Goldenberg, Ryan Paterson, Alan So, Mitchell Humphreys, Claus Roehrborn, Steven Kaplan, Jay Motola, Naeem Bhojani

**Affiliations:** ^1^ University of Montreal Hospital Center University of Montreal Montréal Québec Canada; ^2^ Urology San Diego Clinical Trials San Diego California USA; ^3^ Urology Adult Pediatric Urology & Urogynecology, P.C. Omaha Nebraska USA; ^4^ Urology Virginia Urology Richmond Virginia USA; ^5^ Institute of Urology University of Southern California Los Angeles California USA; ^6^ University Health Network University of Toronto Toronto Ontario Canada; ^7^ Urology Albany Medical College Albany New York USA; ^8^ Urology Indiana University Health Physicians Indianapolis Indiana USA; ^9^ Urology Urology of Virginia Virginia Beach Virginia USA; ^10^ Urology Wake Forest School of Medicine Winston‐Salem North Carolina USA; ^11^ Urology University of Vermont Medical Center Burlington Vermont USA; ^12^ Urology VA Long Beach Healthcare System Long Beach California USA; ^13^ Urology University of British Columbia Vancouver British Columbia Canada; ^14^ Urology Mayo Clinic Arizona Scottsdale Arizona USA; ^15^ UT Southwestern Medical Center, Department of Urology University of Texas Southwestern Dallas Texas USA; ^16^ Urology Icahn School of Medicine at Mount Sinai New York New York USA

**Keywords:** Aquablation, BPH, LUTS, prostate surgery, robotics, urology

## Abstract

**Objective:**

The objective of this study is to determine if Aquablation therapy can maintain its effectiveness in treating men with lower urinary tract symptoms (LUTS) due to benign prostatic hyperplasia (BPH) with large‐volume (80–150 cc) prostates at 3 years.

**Subjects and Methods:**

One hundred one men with moderate‐to‐severe BPH symptoms and prostate volumes between 80 and 150 cc were enrolled in a prospective, nonrandomized, multicenter, international clinical trial in late 2017. Baseline, procedural, and follow‐up parameters were recorded at baseline and scheduled postoperative visits. IPSS, Qmax, and treatment failure are reported at 3 years.

**Results:**

The mean prostate volume was 107 cc (range 80–150). Mean IPSS improved from 23.2 at baseline to 6.5 at 3 years (16.3‐point improvement, *p* < 0.0001). Mean IPSS quality of life improved from 4.6 at baseline to 1.1 at 3 years (improvement of 3.4 points, *p* < 0.0001). Maximum urinary flow increased from 8.7 to 18.5 cc/s. At 3 year follow‐up, 6% of treated patients needed BPH medication and an additional 3% required surgical retreatment for LUTS.

**Conclusions:**

Three‐year follow‐up demonstrates a sustained symptom reduction response along with low irreversible complications to Aquablation in men with LUTS due to BPH and prostates of 80–150 cc. Current treatment options available for men with prostates of this size have similar efficacy outcomes but are burdened with high rates of irreversible complications. There are now numerous clinical studies with Aquablation used in various prostates sizes, and it should be offered as an option to men with LUTS due to BPH.

## 1 INTRODUCTION

Increasing evidence supports the use of robotically executed waterjet‐based resection of the prostate (Aquablation procedure) as an alternative to other tissue resection‐based procedures for men with moderate‐to‐severe lower urinary tract symptoms (LUTS) due to benign prostatic hyperplasia (BPH). Prospective trials have shown excellent safety and effectiveness in men with smaller (30–80 cc)^1^ and larger (80–150 cc) prostates.^2^, ^3^ Postmarket studies have confirmed these findings.^4^, ^5^ Studies have shown persistent improvements in symptoms related to BPH, uroflow measures, and quality of life (QOL). Surgical revision rates have been found to be approximately 1% per year.^3^, ^4^, ^6^ Compared with TURP, the Aquablation procedure has a lower rate of postoperative ejaculatory dysfunction for small (30–80 cc) and large (80–150 cc) prostate glands.^1^, ^7^

Alternative surgical management options for men with larger prostates are limited. Simple open prostatectomy carries increased surgical risks and transurethral resective procedures for large prostates can be very long along with increased risk of bleeding, transfusions, and retreatment. Moreover, while holmium laser enucleation of the prostate (HoLEP) is globally accepted as a gold standard endoscopic treatment for large‐volume prostates, its skillset and practice remain limited to few urologists.^8^, ^9^ By leverage imaging, software, and robotics, Aquablation has standardized the procedure regardless of prostate size that results in a short learning curve and reproducible, consistent operating times.^10^ Herein, we report 3‐year outcomes in men with larger (80–150 cc) prostates who underwent the Aquablation procedure as part of a prospective multicenter clinical trial.

## 2 METHODS

### 2.1 Trial design and participants

WATER II (NCT03123250) is a prospective, multicenter clinical trial conducted at 16 centers in the United States and Canada. Adult men age 45–80 were included if they had a prostate volume between 80 and 150 cc by transrectal ultrasound, baseline IPSS ≥ 12,^11^ a maximum urinary flow rate (Qmax) < 15 ml/s, a serum creatinine < 2 mg/dl, a history of inadequate or failed response to medical therapy and mental capability, and willingness to participate in the study. Men were excluded if they had body mass index ≥ 42 kg/m^2^, a history of prostate or bladder cancer, clinically significant bladder calculus or bladder diverticulum, active infection, previous urinary tract surgery, urinary catheter use daily for 90 or more days, chronic pelvic pain, diagnosis of urethral stricture, meatal stenosis or bladder neck contracture, use of anticholinergic agents, and other general conditions that could prevent adequate study follow‐up. Patients with prior prostate surgery were not excluded. Men with urinary retention were excluded if the catheter was in place for more than 90 days. Each center obtained institutional review board/ethics committee approval prior to study start. Overall, 101 were enrolled in the original study at 16 sites. The 1‐year study was extended during trial follow‐up to include visits annually out to 5 years. Among the treated patients, 86 men agreed to long‐term follow‐up. The study was sponsored by the device manufacturer.

At baseline and selected follow‐up visits, participants completed the following questionnaires: IPSS, Incontinence Severity Index, Pain Intensity Scale, International Index of Erectile Function (IIEF‐15),^12^ the Male Sexual Health Questionnaire (MSHQ‐EjD^13^), uroflowmetry, and postvoid residual (PVR) volume measurements. Scheduled 3‐year follow‐up included PSA, uroflow, and IPSS questionnaires only as well as adverse event assessment. Surgical retreatment was defined as the need for a secondary procedure to treat prostatic tissue for recurrent LUTS.

The Aquablation procedure was performed using the aquabeam System (PROCEPT BioRobotics, Redwood City, California, USA).^14^ Briefly, after induction of general or spinal anesthesia, a 24F single‐use handpiece was inserted into the prostatic urethra and secured into place using a bed‐mounted arm. Using real‐time transrectal ultrasound guidance, the surgeon defined the target anatomic resection contour on a computer console. Contours were selected to avoid damage to the bladder neck, ejaculatory ducts, and urinary sphincter. Furthermore, apical treatment was also planned ipsilaterally to ensure no injury to the verumontanum and its underlying ejaculatory ducts (butterfly cuts). Tissue was then treated utilizing an automated, robotic‐executed, high‐velocity waterjet with up to 2.4‐cm treatment depth. For larger prostates, the Aquablation procedure typically required two treatment passes of the aquabeam probe for larger tissue removal.

Post‐Aquablation, the bladder was irrigated using a 24–27 resectoscope sheath along with a Toomey syringe. Thereafter, hemostasis was delivered via low‐pressure tamponade with a standard three‐way 24 French hematuria Foley catheter inflated to 40–80 cc of saline either at the bladder neck (98 cases) or within the prostatic fossa (three cases), followed by continuous bladder irrigation as previously described, followed by use of the continuous traction device.^15^ The continuous traction device was designed for the specific purpose to hold catheter traction. Catheter traction was held for an average of 18 h in this study. Unlike contemporary Aquablation treatment series,^16^ it is noteworthy that no cases utilized electrocautery for hemostasis.

### 2.2 Data monitoring

All study data were collected using an electronic data capture system. Study data were 100% source‐verified by study monitors.

### 2.3 Statistical analysis

Changes in continuous measures were assessed using *t* tests and/or repeated measures analysis of variance. Exact binomial methods were used to calculate confidence intervals for proportions. All statistical analysis was performed using R,^17^ and a *p* value of <0.05 was considered clinically significant.

## 3 RESULTS

In the original study, 101 men were enrolled at 16 sites (24 surgeons) between September and December 2017. Consent for study extension at all 16 sites was obtained in 86 subjects (85%).

Baseline patient characteristics (*n* = 101) are summarized in Table 1. Mean age was 68 years (52–72) and baseline IPSS was 23 (12–35). Among the treated patients, 16 men (16%) had used a urinary catheter in the 45 days prior to enrollment. Mean prostate volume was 107 cc (80–150). A median lobe was present in 83% of cases with an average intravesical prostatic protrusion (IPP) distance of 1.8 cm (0.7–6.8). Study procedures were performed under general anesthesia in 18% and spinal anesthesia in 82% of cases. Mean operative time (defined as TRUS insertion to urinary catheter placement), Aquablation treatment time, and average number of treatment passes were 55 min (range 25–111 min), 8 min (range 3–17 min), and 1.8 passes (33% 1 pass, 56% 2 pass, and 10% 3 or more), respectively.

**TABLE 1:** Baseline characteristics (*n* = 101)

Characteristic	Statistic
Age, years, mean (SD), range	67.5 (6.6), 52–79
Body mass index, mean (SD), range	28.4 (4.2), 22–41
Race	
Asian	5 (5.0%)
Black	6 (5.9%)
White	88 (87.1%)
Other	2 (2.0%)
Ethnicity	
Hispanic or Latino	9 (8.9%)
Non‐Hispanic or Latino	92 (91.1%)
Prostate specific antigen, g/dl; mean (SD), range	7.1 (5.9), 0.34–29
Use of catheters in 45 days prior to enrollment	16 (15.8%)
Prostate size (TRUS), cc; mean (SD), range	107.4 (22.1), 80–150
Middle lobe	84 (83.2%)
Intravesical component	81 (96.4%)
Intravesical protrusion, mm; mean (SD)	1.8 (0.8)
Baseline questionnaires	
IPSS score, mean (SD), range	23.2 (6.3), 12–35
IPSS QOL, mean (SD), range	4.6 (1.0), 2–6
MSHQ‐EjD,^a^ mean (SD), range	8.2 (3.9), 1–15
SHIM,^a^ mean (SD), range	15.1 (7.4), 2–25

^a^ Sexually active men only.

**FIGURE 1:**
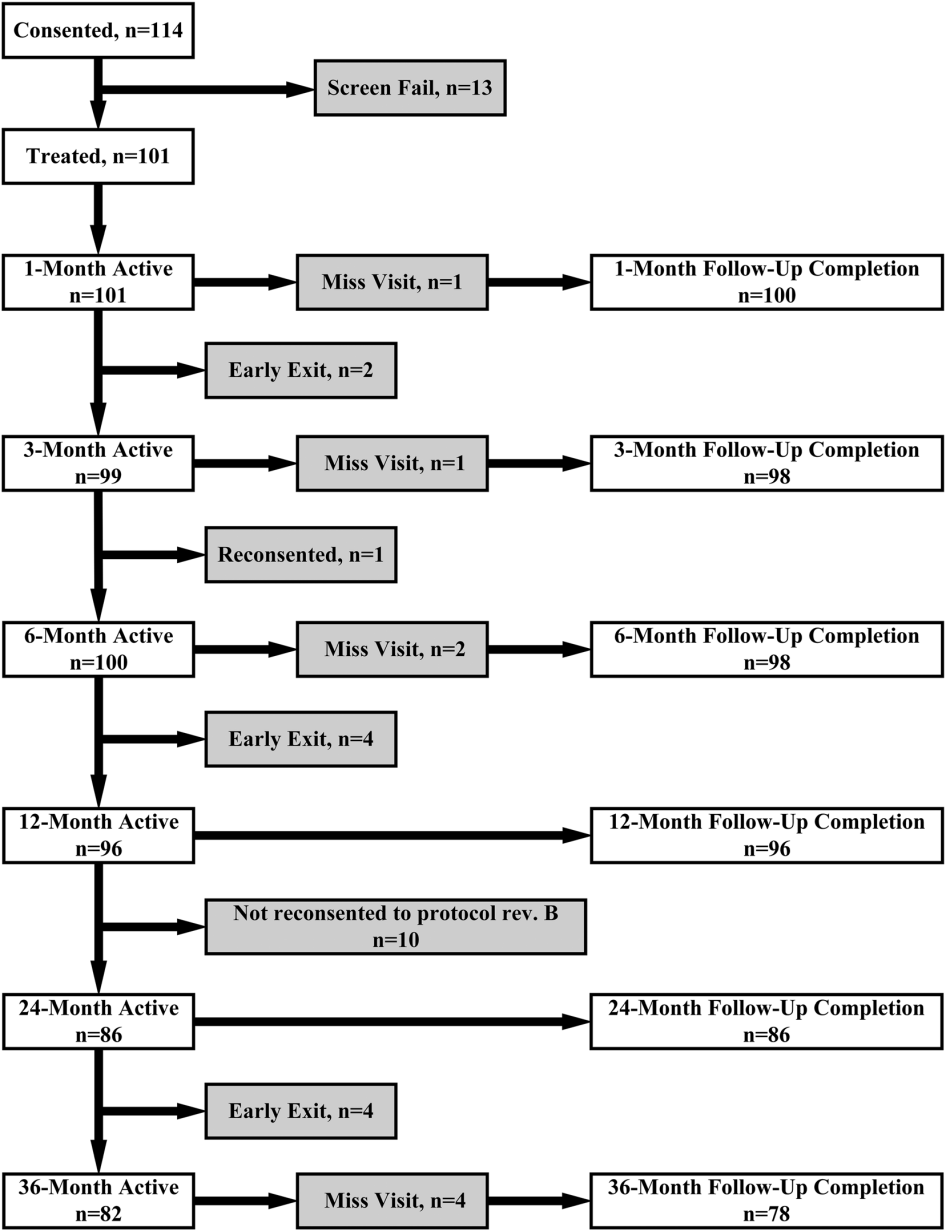
CONSORT diagram

**FIGURE 2:**
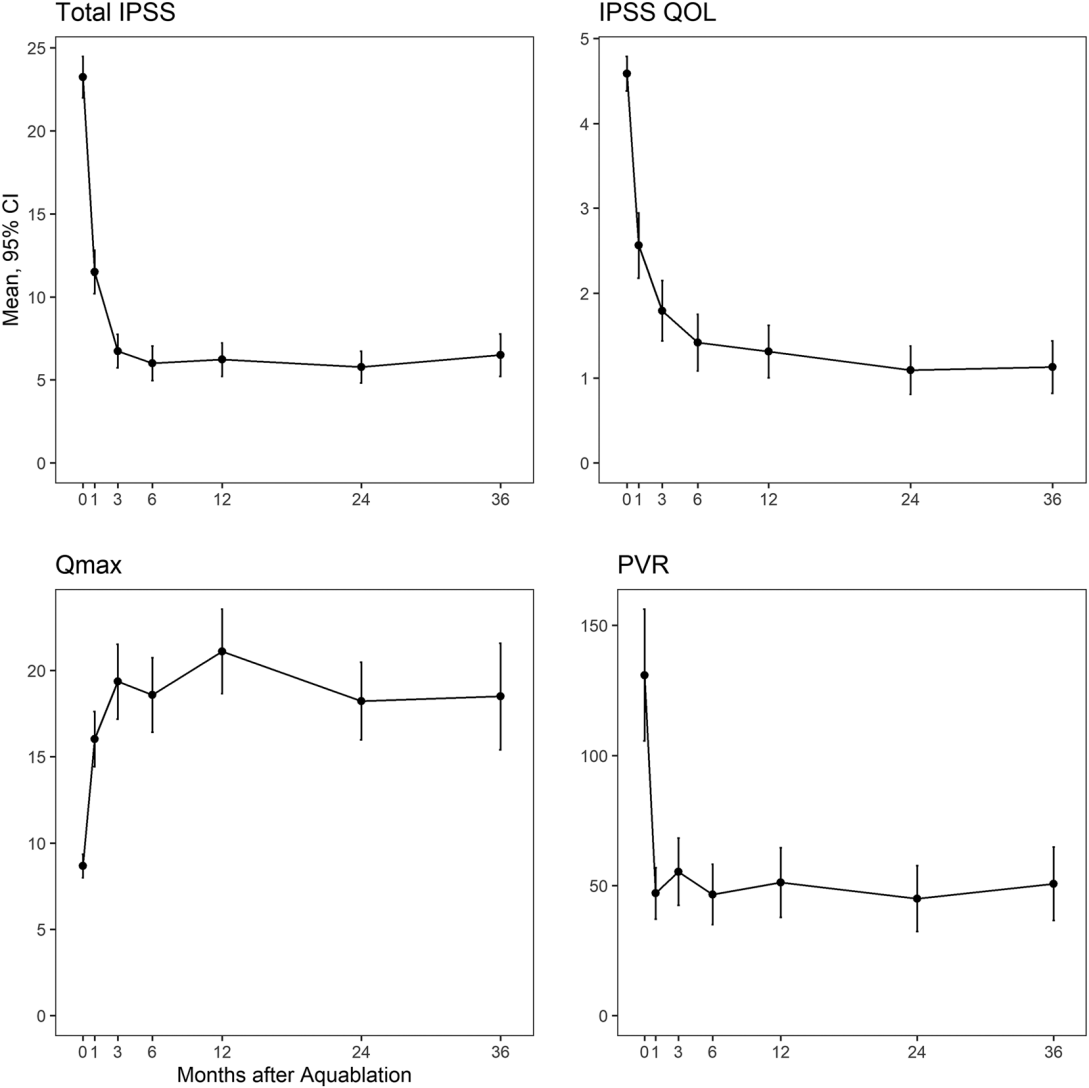
Improvement in IPSS, IPSS quality of life (QOL), Qmax (maximum urinary flow rate, ml/s), and postvoid residual (ml) after Aquablation

Three‐year follow‐up was obtained in 78 subjects (77%) (Figure 1). Follow‐up may have been limited by the COVID‐19 pandemic. Mean (SD) IPSS improved from 23.2 (6.3) at baseline to 6.5 (5.7) at 3 years (a 16.3‐point improvement, *p* < 0.0001, Figure 2). Three‐year IPSS scores were independent of both baseline IPSS and prostate size. IPSS QOL decreased from 4.6 (1.1) at baseline to 1.1 (1.4) at 3 years (a 3.4‐point reduction, *p* < 0.0001). In patients reporting catheter use in the 45 days prior to enrollment, IPSS decreased from 26.3 (7.4) at baseline to 3.7 (2.4) at 3‐year follow‐up. No patient using a catheter prior to surgery has had to return to using a catheter post operatively.

**FIGURE 3:**
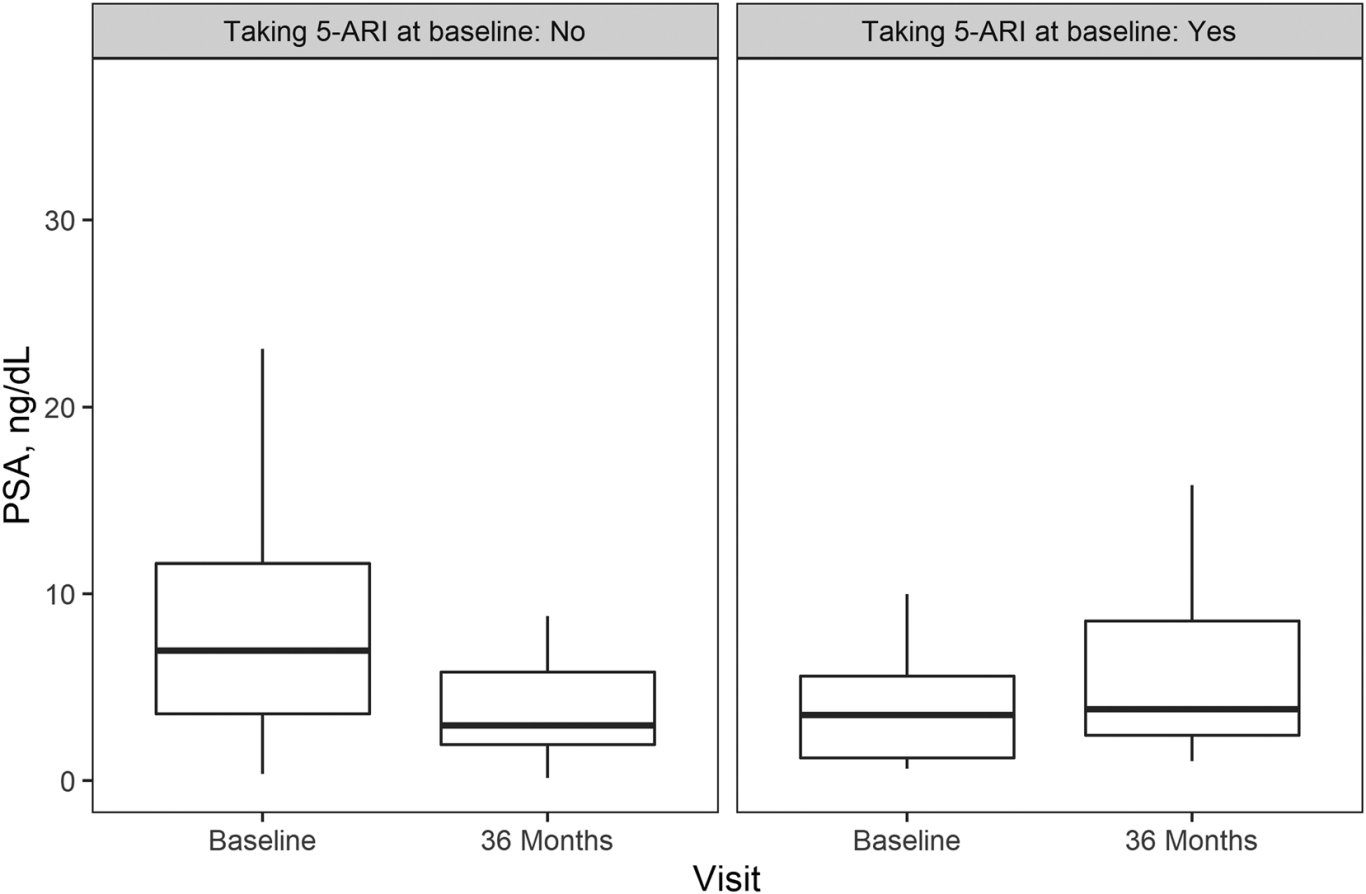
PSA reduction in men grouped by 5‐ARIs status prior to Aquablation

Maximum urinary flow rate increased from 8.7 (3.4) to 18.5 (13.8) cc/s (an improvement of 10.5 cc/s at 3 years, *p* < 0.0001),^15^ which was a sustained outcome achieved since the 3 month visit. PVR urinary volume decreased from 131 (125) cc at baseline to 51 (63) cc at 3 years, which was a sustained outcome achieved since the 1 month visit. Mean (SD) serum PSA decreased from 7.1 (5.9) at baseline to 5.0 (6.0) at 3 years. In men not taking 5‐ARI prior to surgery, PSA was decreased substantially at 36 months (*p* < 0.0001; Figure 3).

At 3‐year follow‐up, 6% of treated patients needed BPH medication and an additional 3% required surgical retreatment for LUTS. After Year 2, no subject underwent a surgical procedure for urethral stricture, bladder neck contracture, or urinary incontinence. The number of urologic events was small with no remarkable difference compared to previous results (Table 2).

**TABLE 2:** Number of events, number of subjects with event, and rate by days since surgery

	0–6 months			6–12 months			12–36 months		
	Events	Subjects	Rate (%Subjects *N* = 101)	Events	Subjects	Rate (%Subjects *N* = 101)	Events	Subjects	Rate (%Subjects *N* = 101)
Bladder stones	‐	‐	‐	3	3	3.0%	‐	‐	‐
Bleeding^a^									
Transfusion, periprocedure	7	6	5.9%	‐	‐	‐	‐	‐	‐
Transfusion, delayed (<30 days)	4	4	4.0%	‐	‐	‐	‐	‐	‐
Transfusion, delayed (>30 days)	‐	‐	‐	‐	‐	‐	‐	‐	‐
Takeback for fulguration without transfusion	3	3	3.0%	‐	‐	‐	‐	‐	‐
Bleeding event not requiring transfusion or takeback	2	2	2.0%	2	2	2.0%	5	5	5.0%
Cardiac	3	3	3.0%	‐	‐	‐	‐	‐	‐
Cerebrovascular accident	1	1	1.0%	‐	‐	‐	‐	‐	‐
Chronic cystitis	‐	‐	‐	‐	‐	‐	1	1	1.0%
Dysuria	3	3	3.0%	‐	‐	‐	‐	‐	‐
Ejaculatory dysfunction	15	15	14.9%	1	1	1.0%	2	2	2.0%
Erectile dysfunction	‐	‐	‐	‐	‐	‐	2	2	2.0%
Hematospermia	‐	‐	‐	1	1	1.0%	‐	‐	‐
Meatal stenosis	4	3	3.0%	‐	‐	‐	‐	‐	‐
Multisystem organ failure^b^	1	1	1.0%	‐	‐	‐	‐	‐	‐
Other: Nonurological	2	2	2.0%	8	6	5.9%	6	4	4.0%
Pain	1	1	1.0%	‐	‐	‐	‐	‐	‐
Prostate cancer	‐	‐	‐	1	1	1.0%	‐	‐	‐
Rising PSA	‐	‐	‐	‐	‐	‐	6	6	5.9%
Skin infection	1	1	1.0%	‐	‐	‐	‐	‐	‐
Urethral stricture	1	1	1.0%	‐	‐	‐	‐	‐	‐
Urinary frequency	2	2	2.0%	‐	‐	‐	5	5	5.0%
Urinary incontinence	6	6	5.9%	1	1	1.0%	1	1	2.0%
Urinary retention	1	1	1.0%	1	1	1.0%	2	2	2.0%
Urinary tract infection	8	7	6.9%	11	8	7.9%	7	5	5.0%
Urinary urgency	2	2	2.0%	2	2	2.0%	3	3	3.0%

*Note*: Adverse events up to 6 month were adjudicated against Clavien–Dindo Grade 1 persistent or higher.

^a^ Hierarchical reporting of bleeding events.

^b^ Due to undiagnosed underlying conditions and not a direct result of Aquablation.

Selected subgroup analysis was performed for patients with moderate symptoms (baseline IPSS score < 20), mean IPSS improved from 16.1 at baseline to 6 at 3 years (improvement of 10 points); for subjects with severe symptoms (baseline IPSS ≥ 20), mean IPSS improved from 26.9 to 6.8 at 3 years, an improvement of 20 points. The difference in 3‐year scores between those with moderate versus severe baseline scores was not significant (*p* = 0.52). There were no differences in other efficacy measures between the subgroups.

Moreover, among the 16 catheter‐dependent subjects within the 45 days prior to eligibility evaluation, IPSS improved from 26.3 at baseline to 3.7 at 3 years. This change was similar to that observed in men without baseline urinary retention. No subject with baseline urinary retention required catheter use following Aquablation. Two subjects (2%) experienced de novo, persistent incontinence requiring pad use following the Aquablation procedure that persisted to 1 year. One of the subjects exited the study at 1 year preventing any further updates on the status. The second patient has reported his incontinence has resolved.

## 4 DISCUSSION

Our study provides strong evidence that the Aquablation procedure provides excellent rapid and sustained mid‐term (3‐year) relief of LUTS related to BPH. Importantly, improvement in BPH symptoms (IPSS, IPSS QOL) and uroflow measures (Qmax and PVR) were immediate, clinically meaningful and sustained to 3 years. Changes were consistent with an earlier randomized trial of Aquablation versus TURP in smaller (30–80 g) prostates^1^ but were especially notable in the current study given the larger prostate size enrolled (enrollment criteria of 80–150 cc, mean 107 cc, 83% with a large median lobe), a group that typically would require a simple prostatectomy or an enucleation procedure with associated risks of incision and transfusions for simple prostatectomy and a very steep learning curve for enucleation. In this early inaugural study, notably without use of any cautery for hemostasis, 5.9% of patients required a transfusion in recovery prior to discharge. No transfusion occurred in the operating room. In both situations of a transfusion or takeback for fulguration, the catheter tension device was not sufficient for complete hemostasis. Moreover, contemporary series of Aquablation advocate use of focal bladder neck cautery. A recently published series by Elterman et al from more than 2000 patients with a mean prostate size of 87 cc (range 20–363 cc) resulted in a transfusion rate of only 0.8% (95% CI 0.5%, 1.3%). These results compared favorably with other surgical approaches in larger sizes where the observed transfusions rates were TURP (4% to 14%), enucleation (0% to 5%), and simple prostatectomy (0% to 24%).^16^ Further to safety‐related outcomes, most incontinence events were acute, described as mixed incontinence, transient, and very few required the use of a pad. There were no late occurrences of incontinence reported in the study.

Improvements in LUTS were clinically important (mean ~17‐point reduction in IPSS, 3.4‐point reduction in IPSS QOL) and concomitant large improvements were seen in uroflow measures (improvement in Qmax of 11 cc/s), both being durable at 3 years. Nearly all men were medication free (94%) at the end of 3 years. Freedom from surgical retreatment was 97% at 3 years. This compares favorably with two datasets published reporting freedom from surgical retreatment. Welk et al reported on 52 748 men undergoing TURP or PVP with an approximated 3‐year freedom from surgical retreatment of 92% and 89%, respectively.^18^ Gilfrich et al reported on 43 041 men undergoing TURP, PVP, enucleation, or open simple prostatectomy with an approximated 3‐year freedom from surgical retreatment of 93%, 89%, 94%, and 96%, respectively.^19^ Most importantly, these datasets include prostates of all sizes. Literature suggests the rates of retreatment are even higher in patients with glands larger than 80 g.^20^

Our study provides convincing 3‐year evidence that the Aquablation procedure is safe, reproducible, and an effective treatment of LUTS related to BPH. More important, it is feasible and effective for the subgroup of large prostates, for which treatment options are limited. For most practicing urologists (>98%) who do not perform HoLEP, Aquablation may be a reasonable choice to avoid the need for open simple prostatectomy. Other advantages demonstrated in this study along with the postmarket study results reported by Bach et al include a short learning curve demonstrated by surgeons going through a hands‐on training prior to the first day of procedures, procedure reproducibility through image guidance and robotic execution, maintenance of ejaculation and erectile function, shorter operative time (less than 1 h), and shorter length of stay (typically a single night stay), all of which are potentially associated with decreased procedure‐related morbidity.^4^ The average patient undergoing a simple prostatectomy will stay 5 days in the hospital.^21^ Regarding operative time in large prostates, Nguyen et al reported data across a broad range of prostates sizes (30–300 cc) and showed Aquablation was the only option that could maintain a consistent operative time under 1 h whereas ThuLEP, HoLEP, GreenLEP, and PVP far exceeded the 1‐h duration particularly in prostate sizes beyond 150 cc.^10^

Advantages of our study include its prospective multicenter design with careful preoperative and scheduled postoperative visits and assessments. Prospective trials of men with large prostates and now 3‐year follow‐up are uncommon. A trial limitation was the lack of a control group, preventing direct comparisons with other treatment approaches. The level of evidence generated by Aquablation is establishing a new standard for LUTS due to BPH. At the time of this manuscript writing, there has not been any other surgical intervention, including MIST, to conduct two FDA clinical studies where at least one of them randomized against the gold standard TURP. From the three core clinical studies (WATER, WATER II, and OPEN WATER), Aquablation has demonstrated TURP‐like efficacy in symptom reduction and uroflow improvement, yet in an unlimited prostate size range which is unattainable by a TURP surgeon. Additionally, the Aquablation efficacy is coupled with a risk profile for irreversible complications (incontinence, erectile dysfunction, and ejaculatory dysfunction) that are comparable with MIST procedures.^22^, ^23^

## 5 CONCLUSION

The Aquablation procedure is a safe and effective, robotically executed and globally reproducible surgical option for the treatment of BPH‐related LUTS in men with large prostate glands with continued durable outcomes at 3 years coupled with efficient operative times, limited hospitalization, and low retreatment rates.

## AUTHOR CONTRIBUTIONS

All authors on the manuscript were involved with recruiting, treating patients, and data collection. Kevin C. Zorn and Naeem Bhojani led the manuscript effort.
